# Monitoring the Influence of Hand, Foot, and Mouth Disease: New Guidelines on Patient Care during the 2011–2012 Multiwaves and Multivariant Outbreak in Hai Phong City, Vietnam

**DOI:** 10.3390/pathogens13090777

**Published:** 2024-09-09

**Authors:** Nghia Ngu Duy, Le Thi Thanh Huong, Patrice Ravel, Le Thi Song Huong, Ankit Dwivedi, Guilhem Kister, Laurent Gavotte, Christian A. Devaux, Vu Dinh Thiem, Nguyen Thi Hien Thanh, Tran Nhu Duong, Nguyen Tran Hien, Emmanuel Cornillot, Roger Frutos

**Affiliations:** 1 National Institute of Hygiene and Epidemiology, 1 Yersin Street, Hanoi 100000, Vietnam; ndn@nihe.org.vn (N.N.D.); huongev.nihe@gmail.com (L.T.T.H.); thiemvd@gmail.com (V.D.T.); enterovirus@nihe.org.vn (N.T.H.T.); trannhuduong@gmail.com (T.N.D.); ngtrhien@yahoo.com (N.T.H.); 2 Institut de Recherche en Cancérologie de Montpellier (U1194), IRCM, Université de Montpellier, Campus Val d’Aurelle, CEDEX 5, 34298 Montpellier, France; patrice.ravel@umontpellier.fr (P.R.); adwivedi@som.umaryland.edu (A.D.); emmanuel.cornillot@umontpellier.fr (E.C.); 3 Hai Phong Preventive Medicine Center, Hai Phong City 180000, Vietnam; nguduynghia@gmail.com; 4 Faculty of Pharmacy, University of Montpellier, 15 av Charles Flahault, BP14491, CEDEX 5, 34093 Montpellier, France; guilhem.kister@umontpellier.fr; 5 Espace-DEV, Université de Montpellier, 500 Rue Jean François Breton, 34090 Montpellier, France; laurent.gavotte@umontpellier.fr; 6 IHU Méditerranée-Infection, 19-21 Bd Jean Moulin, 13005 Marseille, France; christian.devaux@mediterranee-infection.com; 7 CIRAD, UMR 17, Intertryp, TA-A17/G, Campus International de Baillarguet, CEDEX 5, 34398 Montpellier, France; 8 Faculty of Medicine-Ramathibodi Hospital, Mahidol University, Bangkok 10400, Thailand

**Keywords:** HFMD, enterovirus, coxsackievirus, EV-A71, CV-A6, CV-A16, Hai Phong, Vietnam

## Abstract

From 2011 to 2012, Northern Vietnam suffered its first large-scale hand, foot, and mouth disease (HFMD) epidemic. Two sets of official guidelines were issued during the outbreak to handle the HFMD crisis. The city of Hai Phong was used as a model to analyze the impact of the released guidelines. A total of 9621 HFMD cases were reported in Hai Phong city from April 2011 to December 2012. Three distinct waves of HFMD occurred. Enterovirus A71 and Coxsackievirus A16 were successively associated with the epidemics. Two periods, before and after the guidelines’ release, could be distinguished and characterized by different patient patterns. The time to admission and severity changed notably. Guideline publications help the health system refocus on the 0.5–3 years age group with the highest incidence of the disease. The three waves showed different special distribution, but the main routes of infection were rivers and local secondary roads, most likely through local trade and occupational movements of people.

## 1. Introduction

Hand, foot, and mouth disease (HFMD) is an acute febrile illness in children with a papulovesicular skin rash at the palms or soles of the feet, or both. The presentation can be with or without the inclusion of mouth ulcers. HFMD can result in severe complications such as encephalitis, aseptic meningitis, pulmonary edema, myocarditis, and death [1]. HFMD is caused by several types of Enterovirus A, including Coxsackievirus A (CVA) and Enterovirus EV-A71 [2,3]. The EV-A71 viruses are genetically related to CVA and have diverged as recently as the 1920s [4]. Both EV-A71 and CVA infections have been associated with severe HFMD in young children, occasionally resulting in death [1,5,6,7]. The incidence of EV-A71 was over 70% before the development of the anti-HFMD vaccine. With this vaccine being essentially directed against EV-A71, the incidence of EV-A71-related HFMD cases decreased, and most of the cases observed after the introduction of the vaccine were CVA-related [8].

HFMD epidemics pose a challenge for the healthcare system in terms of caring for patients due to (1) the symptoms and their evolution, as explained above, and (2) the sudden influx of large numbers of patients. COVID-19 has shown how health systems and the society itself can be completely disrupted by an epidemic. Monitoring the 2011–2012 HFMD epidemic in Vietnam was interesting in several ways. There is a database of almost 10,000 patients spread over the three waves that affected the Hai Phong region from late 2011 to the end of 2012. The healthcare system, which was severely affected by the first wave, responded in the middle of the second wave with a change in patient management. The previous guidelines for managing HFMD did not consider the severity of the infection. The new guidelines introduced in 2011 described several levels of severity with associated recommendations. Depending on the severity, patients would be immediately hospitalized or treated as outpatients. We show that this change enabled at-risk individuals to be cared for without modifying the monitoring of the epidemic. Although EV-A71 was isolated for the first time in Vietnam in 2003, the first outbreak of HFMD was reported in South Vietnam in 2005 [9]. The 2011 HFMD epidemic was the first one to occur in Northern Vietnam [10].

## 2. Materials and Methods

Epidemiological information and specimen collection. Since 2011, all HFMD cases in Hai Phong city have been reported to the National Institute of Hygiene and Epidemiology (NIHE) through the national communicable disease surveillance system as specified by the published guidelines. HFMD patients who were present at health centers or hospitals were diagnosed and classified into four severity levels (Supplementary Table S1). The evaluation of the disease was performed according to the guidelines specifically published by the Vietnamese Ministry of Health, which are based on, but slightly different from, the WHO and Taiwanese guidelines [1,11].

PCR amplification and nucleotide sequencing. Molecular analyses were performed on 257 throat swabs collected at the main pediatric hospital in Hai Phong City from HFMD-diagnosed patients from 14 out of the 15 districts. From February 2012 through August 2012, following authority requirements, samples were collected only from patients presenting severe symptoms (severity level 2b up). Enterovirus-positive and EV-A71-positive samples were identified according to Nix et al. using *SO*, *AN*, and *MAS* primers [12,13]. Samples collected in November 2011, December 2011, March 2012, and from September 2012 to December 2012 were subjected to Sanger sequencing and analyzed with the Enterovirus Genotyping Tool (http://www.rivm.nl/mpf/enterovirus/typingtool, accessed on 20 October 2023).

Statistical analysis. Population size was estimated using 2009 census data for comparative analysis [14]. Incomplete data (less than 5%) were excluded, leaving 9621 cases for the analysis. Each patient was described by age (date of birth was not available), severity, date of onset of the disease (first fever), date of admission to hospital, and personal address. Hierarchical classification using Gower distance was used to cluster patients as follows: age, time from onset to admission (in days), and severity (as qualitative value). Primary Component Analysis (PCA) was performed using age, gender, district related to the address, time from onset to admission (in days), and severity. Hai Phong districts were numbered from 1 to 14. The severity values were considered in a continuous manner, giving the value 2 to severity 2a and 2.5 to severity 2b. Clustering and graphics were performed with R.3.1. Statistical tests (https://doi.org/10.59350/t79xt-tf203, accessed on 20 October 2023) on clinical data were performed using Stata 9.0 for Windows. The mean comparison was implemented by a Student’s *t*-test. A Chi-square test was used to compare the proportions of the Hai Phong city population, and a one-way ANOVA test was used for the variance analysis.

Bias and Ethics. Training sessions on HFMD case definition and reporting were organized for the staff of the routine surveillance system to enhance the quality and consistency of case reports. According to the guidelines, all health facilities must systematically conduct surveillance and provide information on all cases, which must be recorded online. They must define each case precisely depending on severity and provide case reports to the Provincial Centers for Communicable Disease Control and to NIHE. Samples must be collected during outbreaks in particular cases with severity (level 2b and above) and must be sent to NIHE for PCR identification. This work was conducted following the requirements of the Vietnamese Ministry of Health and under the Law of Communicable Diseases Prevention and Control, which was passed in 2007.

## 3. Results

**Figure 1:**
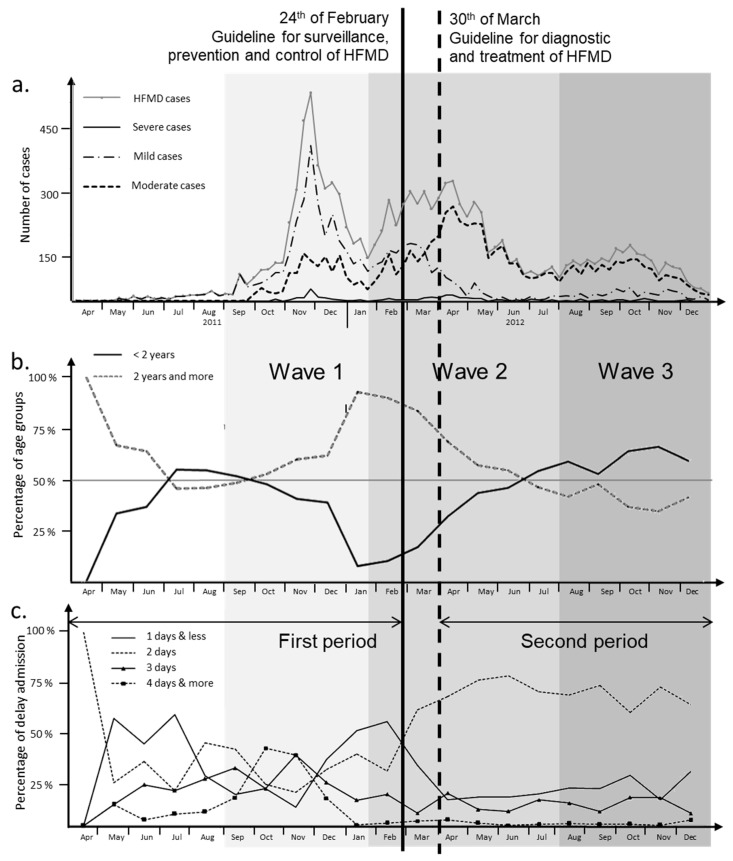
Evolution of HFMD cases and clinical parameters (age, delay of admission, and severity) over the epidemic period (2011–2012). (**a**). Weekly HFMD cases and severity distribution in Hai Phong City (2011–2012) Number of HFMD cases weekly reported to the National Institute of Hygiene and Epidemiology (NIHE), 2011–2012. Each epidemiologic week begins on Monday. Mandatory reporting of the disease began in 2011. Severity levels are based on WHO guidelines for HFMD clinical assessment and case management. Mild cases are cases free of complication (severity score = 1). Moderate cases have a severity score = 2a. Severe cases are characterized by febrile exanthematous symptoms affecting the central nervous system, frequently myoclonus, and more severe neurological complications (severity score = 2b, 3, 4). (**b**). Monthly distribution of age groups of HFMD patients in Hai Phong City (2011–2012). The ages of HFMD patients are divided into two groups: less than 2 years old and 2 years old and above. (**c**). Monthly distribution of delay of admission of HFMD patients in Hai Phong City (2011–2012). Delayed admission is the difference between the date of admission of the patient at the hospital and the date of onset. The distribution shows the proportion of delay of admission for the following classes: one day, two days, three days, or four days and more.

Monitoring of the HFMD burden during the 2011–2012 epidemic. The large HFMD epidemic from 2011–2012 was the first outbreak to occur in Northern Vietnam (65,039 cases). However, the number of cases was higher in the southern part, where HFMD epidemics have been observed since 2005 (157,975 cases). Hai Phong was the hardest hit among the 28 Northern Vietnam provinces during the 2011–2012 HFMD epidemic, with an average prevalence of 524/100,000 persons. A total of 9621 cases were collected during this period from health centers and the main pediatric hospital of Hai Phong City (Supplementary Table S2). The city of Hai Phong is composed of seven urban districts, six countryside districts, and one large island. HFMD cases were reported throughout the entirety of the city, and the epidemic was slightly delayed in 2011 when compared to the rest of Northern Vietnam (Figure 1a). The HFMD epidemic could be subdivided into three separate waves of infection: the first one stretching from August 2011 to January 2012 (Wave 1), the second from February 2012 to July 2012 (Wave 2), and the third one from August 2012 to January 2013 (Wave 3). Before the first wave started, HFMD occurred sporadically in all parts of the city with low incidence (8 cases per week on average). The number of cases increased suddenly in mid-September 2011. The outbreak peaked at 472 cases per week in early December 2011, followed by two smaller peaks in April and October 2012 (Figure 1a and Supplementary Table S3). Two periods, corresponding to different epidemiological patterns, could be distinguished: from August 2011 to March 2012 and from March 2012 to January 2013. The limit between the two periods is marked by the publication of two specific guidelines by the Ministry of Health (MoH). The first one, published on 24 February 2012, concerned surveillance, prevention, and control of HFMD. The second guideline, issued on 30 March 2012, addressed diagnosis and treatment. Only patients with moderate (level 2a) to severe symptoms (level 2b and above) would be taken in charge by the health system (Supplementary Table S1). The evaluation process of the disease burden was, therefore, changed during Wave 2. Moderate forms (severity level 2a) were reported for the majority of cases (5262 cases, 54.92%), but 218 patients were displaying severe symptoms (2.28%). Among this group, only nine patients displayed a severity score of 3, and no cases with the highest level of 4 were recorded (Supplementary Table S2). Gender was not associated with severity (Supplementary Table S4). Moderate forms of HFMD were particularly pronounced in children below 2 years old (*p* < 0.01, Supplementary Table S4). The level of moderate cases was significantly lower during Wave 1 (*p* < 0.01, Supplementary Tables S5 and S6). According to guidelines, the level of moderate cases was significantly higher during the second period. Conversely, the level of mild cases decreased notably to significantly lower after the first period of the epidemic (Figure 1a) (*p* < 0.01, Supplementary Table S7).

The patients’ ages ranged from 24 days to 15 years (median at 2 years, IQR of 2 years, Supplementary Table S2). Out of 9142 cases, 8857 (96.9%) were under the age of 5, with the age-specific incidence being the highest in the 1–2 years age group (3067 cases, 33.6%). It remained very low for older children. The lowest incidence was observed in infants < 5 months (1.88%) and children above 10 years old (0.4%). Boys had a significantly higher prevalence rate (59.74%). Wave 1 was associated with a higher number of children between 2 and 5 years old. Variations in patient age after guideline release were noticeable (Figure 1b). The proportion of cases below 2 years of age was significantly higher at the end of the second period (*p* < 0.01, Supplementary Table S7) and during Wave 3 (*p* < 0.01, Supplementary Table S3). The time after onset to admission was a specific epidemiological parameter used in the present study to monitor the quality of patient care. It varied greatly over the first period (Figure 1c, Supplementary Table S6). The curves for time to admission after onset to admission corresponding to 1-days and 2-days crossed in March 2012 (Figure 1c) concomitantly with those representing mild and moderate levels of severity (Figure 1a).

**Figure 2:**
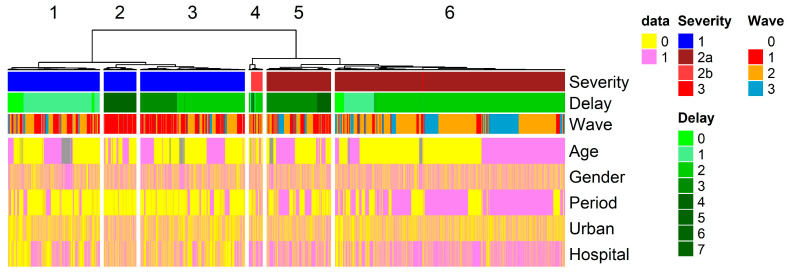
Clusters of patients. The 9621 patients were clustered based on age, the time between the onset of the disease and admission (Delay), and severity. The heatmap represents variables as Boolean values (yellow color for 0 and pink for 1). The pink color corresponds to patients below 2 years of age, male patients, patients from Period 2, patients living in urban areas, and patients registered at the Hai Phong City Pediatric Hospital. The grey color is the unavailable data. The top annotation provides information on the severity, delay of admission, and epidemic waves.

Patient categories. Severity score, epidemic waves, and time from onset to admission were compared with patient classification based on the patient’s age and gender, the period of the study, and the geographic origin of the patient. Six groups (clusters) of patients were identified through hierarchical classification (Figure 2), and each cluster could be associated with the specific parameters defined above. Cluster 1, 2, and 3 gather patients with mild symptoms. These patients were mainly found during Waves 1 and 2 from period 1. The publication of the new guidelines drastically modified the distribution of patients, with patients from Clusters 1, 2, and 3 almost absent from period 2 (Figure 1c and Figure 2). The health system communication policy was active after Wave 1, encouraging parents to keep sick children with mild symptoms at home. The publication of guidelines removed these patients from the statistics, recording only cases with moderate to severe symptoms. As a result, patients in Clusters 5 and 6, who are predominantly associated with moderate symptoms, were mainly associated with the second period. Cluster 4 was associated with patients presenting severe symptoms. The time between the first fever (onset) and the admission to the hospital (delay to admission) was the second variable associated with specific clusters. Cluster 2’s patients with delay over three days were restricted to the first period of the epidemic. Delay to admission was also separating Cluster 5 from Cluster 6. Patients with moderate symptoms were presenting shorter time from onset to admission during period 2. Gender and geographic areas had no relationship with the clustering. Hai Phong City Pediatric Hospital could be associated with Clusters 5 and 6.

Evolution of HFMD admission at Hai Phong City Pediatric Hospital. The proportion of moderate and severe cases admitted at the pediatric hospital increased significantly during the second period (Supplementary Table S8). Concomitantly, the number of HFMD admissions increased in district hospitals. The number of outpatients (treatment at home) also increased notably, with a mild level of severity during the second period. The ratio between young (below 2) and old patients admitted at the pediatric hospital was reversed after March 2012, but not in district hospitals and local health stations (Supplementary Table S9). The share of patients between the pediatric hospital and the local health facilities clearly improved during the second period, with more people from non-urban areas going to district hospitals (Supplementary Table S10). The number of patients admitted at the pediatric hospital coming from non-urban districts compared to urban ones remained the same over the two periods, but the number of non-urban district patients with severe symptoms admitted at the pediatric hospital increased (Supplementary Table S11).

**Figure 3:**
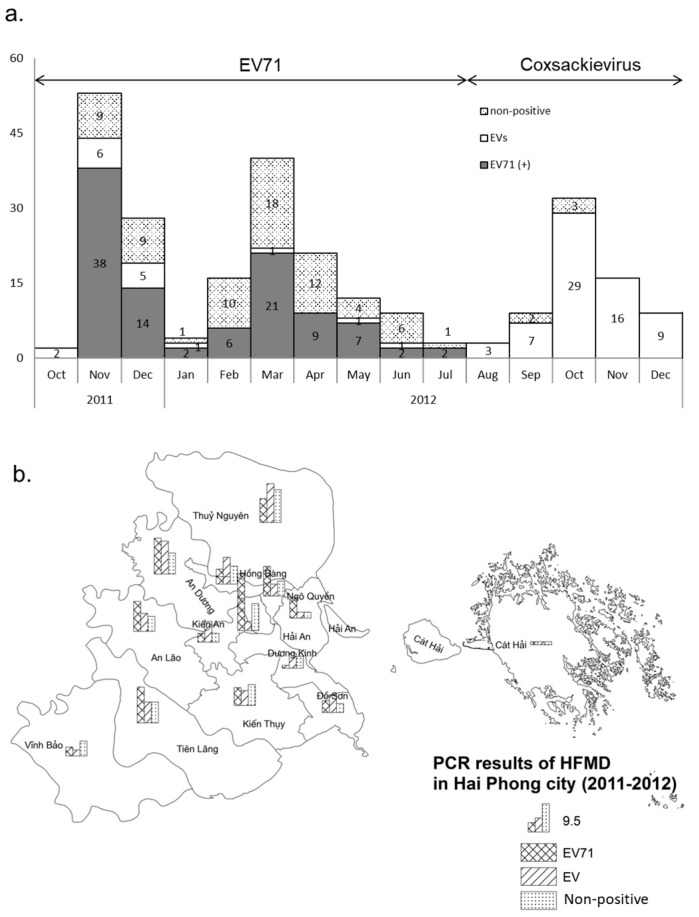
Spatio-temporal distribution of the PCR identifications in Hai Phong. EVs represent all positive results with Enteroviruses using semi-nested PCR. EV-A71 represents all positive results with EV-A71 using semi-nested PCR. “Non-positive” represents all negative results with Enteroviruses using semi-nested PCR. (**a**). Time flow of the 257 PCR identification of HFMD epidemic in Hai Phong. (**b**). Spatial distribution of the 257 PCR identifications in Hai Phong city. Spread of the disease in Hai Phong city. PCA analysis suggested that the distribution among districts was highly variable over the three waves (Supplementary Figure S2). Time differences in the evolution of the epidemic among districts could result from such variation. Early cases appeared in the northern and urban zones of the city (Supplementary Figure S3) and expanded to the west and to the south (Figure 3). Each wave displayed a different main site of emergence (Figure 3, Supplementary Table S12). Wave 1 started in the city center, whereas Waves 2 and 3 emerged at the periphery. The order of occurrence of the first case for each wave defined five groups of districts (Supplementary Figure S3): Hai Phong city center (Group 1), New urban areas in the southern part of the city, and a western rural district (Group 2), peripheric districts and Do Son (Group 3), two rural districts not connected to the main road network (Group 4) and Cat Hai islands (Group 5). The disease’s diffusion to the south followed the axis supported by two main roads, allowing the crossing of rivers and canals. No direct transmission of the disease was observed between the city center and these new urban areas during Waves 2 and 3 (Figure 4). Patterns of transmission among groups were similar for the three waves. The re-emergence of the disease during Waves 2 and 3 shows similarity despite the presence of different etiological agents.

Both EV-A71 and CV-A were present during the epidemic. Molecular diagnostic confirmation was conducted by PCR on 257 samples from cases clinically identified as HFMD. Nearly 71% were positive for Human Enterovirus (182/257). Of the 182 positives, 101 (55%) were EV-A71, and 81 (45%) corresponded to other enteroviruses (EVs) (Figure 3a). The identified EV-A71 isolates belonged to subgenogroup C4, present in the northern and central cities, and C5, also present in the northern, central, and southern cities (Figure 3b). A significant part of patients diagnosed as HFMD during Waves 1 and 2, i.e., 75, were not positive for enterovirus (Figure 3a). EV-A71 coincided with Wave 1 and Wave 2 (Figure 3). Wave 3 was associated with the co-circulation of CV-A6 and CV-A16/(Supplementary Figure S1). The rate of EV-negative samples started to increase in December 2011 and reached a maximum in March 2012; however, this rate was low during Wave 3. This suggests that unknown viruses may have circulated during Waves 1 and 2 but not during Wave 3, which was almost exclusively associated with CV-A6 and CV-A16.

## 4. Discussion

The first HFMD outbreak in North Vietnam. At that time, the 2011–2012 HFMD epidemic was the largest to have ever occurred in Vietnam and the first recorded in the northern part of the country, while Hai Phong city experienced the highest HFMD incidence in North Vietnam. However, no fatal cases were reported in Hai Phong, unlike in South Vietnam [10]. Age-specific incidence was the highest in the 1–2 years age group. This would be in agreement with both the persistence of maternally-derived neutralizing antibodies for up to 6 months and the kinetics of seroprevalence of EV-A71 virus neutralizing antibodies, which increases with age [15,16]. However, this was the very first recorded outbreak of HFMD in northern Vietnam, questioning thus the existence of maternally-derived neutralizing antibodies or pre-existing immunity. Children under 3 years old represented 85.85% of cases. They are in Vietnam traditionally cared for at home by family members. The high HFMD incidence in this population may thus have resulted from contact with adults and older children acting as asymptomatic carriers of the virus [17,18].

Guidelines positively influenced disease management. The first guideline, published on 24 February 2012, was related to surveillance, prevention, and control of HFMD. The second guideline, published on 30 March 2012, addressed diagnosis and treatment and gave a clear HFMD case definition, reporting procedure, and strategy for collecting clinical samples. The first effect of the release of this guideline was a significant increase in the severity score. Indeed, 73.41% of patients scored 2a after guidelines publication, compared to only 25.59% before. The number of moderate and severe cases admitted to Hai Phong Pediatric Hospital increased significantly after the guidelines were published, while the proportion of mild cases decreased sharply. An explanation might be that the release of the guideline influenced the behavior of parents and physicians. Many non-severe cases were most likely declared severe to ensure that the patients would be hospitalized and receive better treatment and monitoring. Another positive effect was the reduced delay between onset and admission after the publication of the guidelines. It decreased during the second period and remained very homogeneous. The presence in nine out of ten clusters in the first half of the outbreak supports this conclusion. The most important feature of the second guideline was the decentralization and transfer of responsibility to healthcare facilities. A more homogeneous spatial distribution of patients visiting pediatric hospitals was visible. Mild cases were treated at the commune level, whereas districts were in charge of moderate cases. At the province level, all cases were addressed. All patients recorded as severe went to province hospitals during the second period, while local health facilities hosted patients unable to go to main hospitals. The patients who remained at home only displayed mild symptoms.

Awareness and legal framework. This positive effect of guidelines is not only the consequence of the publication of guidelines but also of increased awareness and precautious approach from parents and physicians, leading to patients being declared with severe symptoms in order to ensure better treatment and surveillance. This could explain why a higher disease severity score was observed in CV-A-infected patients (Wave 3) than in EV-A71 cases (*p* < 0.01). Awareness led to the modification of guidelines, but changes occurred only after publication, suggesting that the legal framework created by the guidelines was needed for implementation even though awareness was present. Public and professional awareness is not sufficient for implementing changes. Furthermore, the emergence of CV-A (Wave 3) during the second period did not lead to variation in severity and time to admission. This can be easily explained by the fact that Wave 3 occurred after the release of the guidelines. Therefore, the apparent severity is most likely not that caused by the virus. It is rather the consequence of the overstated diagnosis recorded by physicians to ensure the hospitalization of patients. The publication of guidelines during Phase 2 led to different patient patterns, although the virus was the same. The evolution of clinical patterns should not be considered only in light of the evolution or replacement of pathogens or host-pathogen interactions but also according to the evolution of behavior and social perception.

Shift of etiology. Improvement of molecular diagnostics was not considered by the new guidelines, and therefore, they had no impact on the detection of etiological agents in patients. During the Hai Phong outbreak, circulation of both EV-A71 and CV-A was recorded, a feature already reported [9,19,20,21,22]. EV-A71 virus is considered to be the most frequent cause of severe HFMD disease, although CV-A has been shown to cause severe infections with meningitis [11,23,24,25]. An uncharacterized virus might also have circulated during the first wave and mostly the second wave. The high number of EV-non-positive PCR reactions on clinically positive samples suggests that the set of primers used for enterovirus detection might not have been discriminative enough. The ratio of non-positive tests was similar to those previously reported [26,27,28,29]. EV-A71 detection with MAS primers should thus be systematically performed on SO primer products, and the SO222 primer should be redesigned to match the 5′ part of the AN88 primer used for EV detection. More attention should, therefore, be paid to PCR-negative patients.

Spatio-temporal dynamic and disease control. Nguyen et al. have shown the presence of HFMD in provinces west of Hai Phong after the outbreak started in South Vietnam, making the northwestern side of Hai Phong the most likely route of entry [10]. However, despite the main economic role of Hai Phong, no early cases occurred along or at the end of the main highway linking Hai Phong to the rest of the country, indicating that major industrial and export commercial movements are not linked to the dynamic of the disease. Instead, the disease seems to have expanded following the eastbound river system to reach densely populated settlements from where it secondarily expanded through local roads. Disease expansion might thus have followed secondary local commercial routes. These commercial routes allow time for the disease to be transmitted and involve a lot of favorable human-to-human contacts. The presence of early cases on the island and in isolated coastal localities in the southern part of the city also illustrates the role of sea transportation and the role of local trade and occupational activities in the spread of the disease. The southern part may have been affected later due to the fragmentation of the territory and the isolation of the communes by the complex river system. The early occurrence of the disease in northwestern communes not connected to the main local road might be related to specific occupational activities. Considering the average age of the patients (around 2 years of age), the source of contamination must be sought within the asymptomatic adults contaminated during their occupational activities and in local and regional movements.

## Figures and Tables

**Figure 4:**
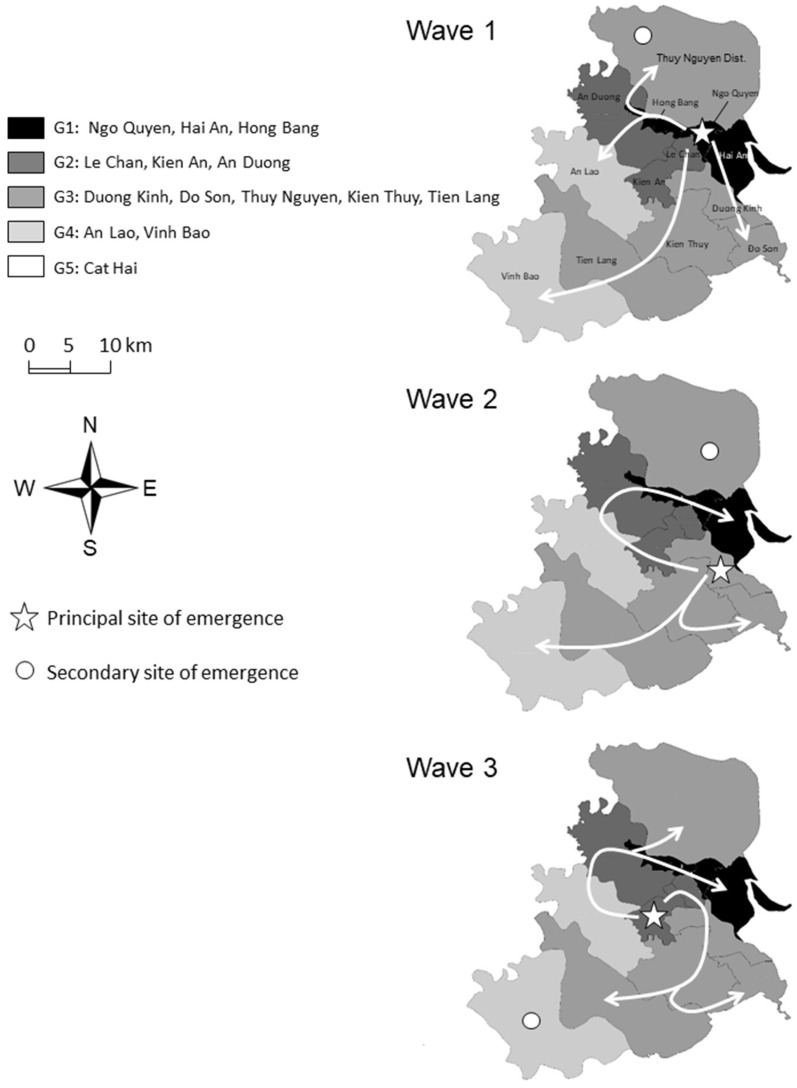
Sites of emergence and expansion of the three waves of HFMD in Hai Phong city during the 2011–2012 epidemics. Shades of grey represent the five groups of districts according to Supplementary Table S12. The island of at Cat Hai is not represented on the map.

## Data Availability

All data are provided in the manuscript and Supplementary Materials.

## Supplementary Materials

The following supporting information can be downloaded at https://www.mdpi.com/article/10.3390/pathogens13090777/s1, Supplementary Figure S1. Spatial distribution of the Coxsackievirus-positive PCR samples during Wave 3. Information was confirmed by Sanger sequencing. Supplementary Figure S2. Primary component analysis. Axis 1 represented more than 95% of the variance associated with patients, based on age, gender, district related to the address, time from onset of symptoms to admission (delay), and severity. Supplementary Figure S3. Propagation of the HFMD epidemic among Hai Phong city districts according to median case. Rural and urban districts were differentiated (Type) and described according to major features (Supplementary Table S12). Stratification (Group) was performed according to the relative order of median in the three waves. The date of the median case and the relative order of the district were given for each wave. Supplementary Table S1. Severity levels of HFMD cases according to guidelines from the Vietnamese Ministry of Health. Supplementary Table S2. Characteristics of reported HFMD cases in Hai Phong city, 2011–2012. Supplementary Table S3: Age of HFMD patients by epidemic waves in Hai Phong City (2011–2012). Supplementary Table S4: Gender, age, living area, pathogen, delay of admission of HFMD reported cases by severity in Hai Phong city, 2011–2012. Supplementary Table S5. Age groups and severity of reported HFMD cases by epidemic outcomes in Hai Phong City between 2011 and 2012. Supplementary Table S6. Gender, Severity, Living area and Delay of admission of reported HFMD cases by epidemic waves in Hai Phong city (2011–2012). Supplementary Table S7. Age groups, severity, delay duration, and living area of reported HFMD cases by epidemic period in Hai Phong City between the first and second study periods. Supplementary Table S8. The severity of reported HFMD cases at the place of admission by epidemic periods in Hai Phong City between the first and second study periods. Supplementary Table S9. Age groups from reported HFMD cases at the place of admission in Hai Phong City during the two periods of the epidemic. Supplementary Table S10. Living area of reported HFMD cases at the place of admission in Hai Phong city. Supplementary Table S11. Living area and severity of reported HFMD cases in Hai Phong Pediatric Hospital City. Supplementary Table S12. Propagation of the HFMD epidemic among Hai Phong city districts according to median case.
