# Supplementary material for: Monitoring the Influence of Hand, Foot, and Mouth Disease: New Guidelines on Patient Care during the 2011–2012 Multiwaves and Multivariant Outbreak in Hai Phong City, Vietnam
Source: Pathogens. 2024 Sep 9;13(9):777. doi: 10.3390/pathogens13090777 (PMC11435139; doi:10.3390/pathogens13090777)
Supplement: Supplementary file 1 [file pathogens-13-00777-s001.zip › pathogens-3112717-supplementary.pdf]

<u>Supplementary Table 1. Severity levels of HFMD cases according to guidelines from the Vietnamese Ministry of Health</u>

Level 1: Only mouth ulcers and/or skin lesions

Level 2a: have in addition one of any signs below:

- Startled in history less than 2 times/30 min and no startled at examination.
- Fever over 2 days or temperature of patient over 39ºC, vomiting, insomniac, irritability

Level 2b**:** Have in additions signs from group 1 or group 2 below:

* Group 1: have one of any signs below

- Startled when examination.
- Startled in history ≥ 2 times /30 min.
- Startled in history together with one sign below:
    - o Drowse
    - o Pulse > 130 times/min (child in the rest, no fever).

* Group 2: have one of any signs below

- High fever ≥ 39,5ºC (anal) and no response to fever release drug.
- Pulse > 150 times /min (child in the rest, no fever).
- Ataxia (locomotor ataxy), tremors
- Nystagmus, squinting eye
- Limb weakness of limb paralysis
- Cranial nerve paralysis

-

Level 3: Have in addition the following signs/symptoms

- Pulse > 170 time/min (child in the rest, no fever).
- Some cases with slow pulse (very severe sign).
- Sweating, coldness of partial or whole body.
- Systolic blood pressure (SBP) increases
    - Child under 12 months of age: SBP > 100 mmHg.
    - Child from 12 to 24 months of age: SBP > 110 mmHg.
    - Child more than 24 months of age: SBP > 115 mmHg.
- Respiratory abnormalities (tachypnea, abnormal breathing)
- Perceptual disorders (dysaesthesia): Glasgow < 10 scores.
- Increase muscle tone.
-

Level 4: have in addition, one of any signs below

- Shock
- Acute pulmonary edema
- Cyanosis, SpO2 < 92%.
- Suspension of breathing, breathing step

Supplementary Table 2. Characteristics of reported HFMD cases in Hai Phong city, 2011-2012

Characteristics	HFMD cases in Hai Phong city during			Children(< 14 years old) in Hai Phong city (%)	*p* value
	2011 (n=3029) n (%)	2012 (n=6592) n (%)	Total (n=9621) n (%)		
Gender					
girls	1,162(38.36)	2,711(41.13)	3,873(40.26)	186.445 (47)	
boys	1,867(61.64)	3,881(58.87)	5,748(59.74)	206.291 (53)	$p< 0.01$
Age (year)					
median (IQR)	2 (2)	2 (2)	2 (2)	NA	
< 0.5	71(2,41)	101(1.63)	172 (1.88)	NA	
0.5 - 1	726 (24.60)	2109 (34.08)	2835 (31.02)	NA	
1-2	970 (32.87)	2097 (33.88)	3067 (33.56)	NA	
2-3	629 (21.31)	1143 (18.47)	1772 (19.39)	NA	
3-4	283 (9.59)	407 (6.58)	690 (7.55)	NA	
4-5	139 (4.71)	180 (2.91)	319 (3.49)	NA	
5-10	119 (4.03)	129 (2.08)	248 (2.71)	NA	
>10	14 (0.47)	23 (0.37)	37 (0.40)	NA	NA
Severity*					
mild	2109 (69.63)	1993 (30.41)	4102 (42.81)	NA	
moderate	849 (28.03)	4413 (67.34)	5262 (54.92)		
severe	71 (2.34)	147 (2.24)	218 (2.28)	NA	NA
fatal	0	0	0		
Living area					
not urban	1474 (48.66)	4080 (61.89)	5554 (57.73)	177.518 (0.55)	
urban	1555 (51.33)	2512 (38.11)	4067 (42.27)	215.219 (0.45)	$p<0.01$
Pathogen					
Non EV-A71	13 (20)	68 (58.12)	81(44.51)	NA	
EV-A71	52 (80)	49 (41.88)	101 (55.49)	NA	$p<0.01$

p value (Chi-square test); NA: not available

*Mild cases correspond to uncomplicated disease (severity level =1).
*Moderate cases correspond to uncomplicated disease (severity level =2a).
*Severe cases correspond to more complicated forms of the disease (severity level = 2b,3 or 4).

Supplementary Table 3: Age of HFMD patients by epidemic waves in Hai Phong City (2011-2012)

Age groups of HFMD cases	Wave 1	Wave 2	Wave 3	*p* value
under 2 years	1150 (34.57)	907 (25.41)	1191 (59.11)	$p < 0.01$
2 years & more	2177 (65.43)	2663 (74.59)	824 (40.89)	
Total	3327 (100%)	3570 (100%)	2015 (100%)	

p value (Chi-square test)

Supplementary Table 4: Gender, age, living area, pathogen, delay to admission of HFMD reported cases by severity in Hai Phong city, 2011 - 2012.

Characteristics		Mild cases	Moderate cases	Severe cases	*p* value
Gender	female	1680 (40.96)[b]	2094 (39.79)	86 (39.45)	$p = 0.50$
	male	2422 (59.04)	3168 (60.21)	132 (60.55)	
Age	>= 2 years	2838 (69.19)	3023 (57.45)	142 (65.14)	$p < 0.01$*
	< 2 years	1264 (30.81)	2239 (42.55)	76 (34.86)	
Living area	not urban	2431 (59.26)	2976 (56.56)	125 (57.34)	$p < 0.05$*
	urban	1671 (40.74)	2286 (43.44)	93 (42.66)	
Pathogen	Non EV-A71	54 (78.26)	15 (19.23)	12 (34.29)	$p < 0.01$*
	EV-A71	15 (21.74)	63 (80.77)	23 (65.71)	
Delay of admission	> 1 day	2451 (59.75)	4551 (86.49)	193 (88.53)	$p < 0.01$*
	= < 1 day	1651 (40.25)	711 (13.51)	25 (11.47)	

p value (Chi-square test)

Mild cases correspond to uncomplicated disease (severity level =1).
Moderate cases correspond to uncomplicated disease (severity level =2a).
Severe cases correspond to more complicated forms of the disease (severity level = 2b,3,4).

Supplementary Table 5. Age groups and severity of reported HFMD cases by epidemic outcomes in Hai Phong city between 2011 and 2012

Severity	Wave 1			Wave 2			Wave 3		
	Age of HFMD patient			Age of HFMD patient			Age of HFMD patient		
	under 2 years	2 years & more	*p* value	under 2 years	2 years & more	*p* value	under 2 years	2 years & more	*p* value
Mild	727 (63.33)	1492 (68.76)	$P < 0.05$	203 (22.43)	1027 (38.98)	$P < 0.01$	190 (15.95)	174 (21.12)	$P < 0.01$
Moderate	387 (33.71)	636 (29.31)		683 (75.47)	1528 (57.99)		981 (82.37)	635 (77.06)	
Severe	34 (2.96)	42 (1.94)		19 (2.1)	80 (3.04)		20 (1.68)	15 (1.82)	
Total	1148 (100%)	2170 (100%)		905 (100%)	2635 (100%)		1191 (100%)	824 (100%)	

p value (Chi-square test)

Mild cases correspond to uncomplicated disease (severity level =1).
Moderate cases correspond to uncomplicated disease (severity level =2a).
Severe cases correspond to more complicated forms of the disease (severity level = 2b,3,4).

$p < 0.05$, indicating that the severity distribution differs significantly between both age groups

Supplementary Table 6. Gender, Severity, Living area and Delay of admission of reported HFMD cases by epidemic waves in Hai Phong city (2011-2012)

Epidemic waves	Gender			Severity				Living area			Delay of admission		
	Male	female	Test*	mild	moderate	severe	Test§	urban	rural	Test*	mean	SD	test#
All period	5748 (59.74)	3874 (40.26)	*p*< 0.01	3813 (42.97)	4850 (54.66)	210 (2.37)	NA	4067 (42.27)	5554 (57.73)	*p*< 0.01	2.08	1.06	NA
Wave 1	2023 (60.81)	1304 (39.19)	*p*< 0.01	2219 (66.88)	1023 (30.83)	76 (2.29)		1628 (48.93)	1699 (51.07)	*p*< 0.01	2.47	1.37	*p*< 0.01
Wave 2	2063 (57.79)	1507 (42.21)	*p*< 0.01	1230 (34.75)	2211 (62.46)	99 (2.8)	*p*< 0.01	1286 (36.02)	2284 (63.98)	*p*< 0.01	1.85	0.79	
Wave 3	1227 (60.89)	788 (39.11)	*p*< 0.01	364 (18.06)	1616 (80.2)	35 (1.74)		838 (41.59)	1177 (58.41)	*p*< 0.01	1.87	0.73	

*: Chi2 one sample, proportion comparison with whole population (male rate = 0.53 and urban rate = 0.45)

§: Chi2 test for proportion comparison of multi groups

#: one-way ANOVA test for mean comparison of multi groups

Mild cases correspond to uncomplicated disease (severity level =1).
Moderate cases correspond to uncomplicated disease (severity level =2a).
Severe cases correspond to more complicated forms of the disease (severity level = 2b,3,4).
p < 0.001, indicating that the severity distribution differs significantly between the three waves

Supplementary Table 7. Age groups, severity, delay duration and living area of reported HFMD cases by epidemic period in Hai Phong city between first and second study period.

Epidemic periods	*Age groups*			*Severity*				*Delay duration*					*Living area*		
	under 2 years	2 years & more	*p* value	mild	moderate	severe	*p* value	1 day & less	2 days	3 days	4 days & more	*p* value	*Not urban*	*urban*	*p value*
1st period	1337 (39.30)	2850 (55.93)	$p <$ 0.01	2817 (80.28)	1271 (25.59)	88 (44.44)	$p <$ 0.01	1265 (63.44)	1118 (27.14)	1036 (66.71)	768 (92.42)	$p <$ 0.01	2168 (45.91)	2019 (53.47)	$p <$ 0.01
2nd period	2065 (60.70)	2246 (47.07)		692 (19.72)	3509 (73.41)	110 (55.56)		729 (36.56)	3002 (72.86)	517 (33.29)	63 (7.85)		2554 (54.09)	1757 (46.53)	
Total	3402 (100%)	5096 (100%)		3509 (100%)	4780 (100%)	198 (100%)		1994 (100%)	4120 (100%)	1553 (100%)	831 (100%)		4722 (100%)	3776 (100%)	

p value (Chi-square test)

Mild cases correspond to uncomplicated disease (severity level =1).
Moderate cases correspond to uncomplicated disease (severity level =2a).
Severe cases correspond to more complicated forms of the disease (severity level > 2b,3,4).
1st period: first half of the epidemic (Apr 2011 – 24th Feb 2012).
2nd period: second half of the epidemic (30th Mar 2012 – 31th Dec 2012).

Supplementary Table 8. Severity of reported HFMD cases at place of admission by epidemic periods in Hai Phong city between first and second study period.

	Commune health station				*District hospital*				*Hai Phong Pediatric hospital*				*At home (outpatient)*			
	mild	moderate	severe	*sum*	mild	moderate	severe	*sum*	mild	moderate	severe	sum	mild	moderate	severe	*sum*
1st period	4 (67)	-	2 (34)	6 (100)	508 (93)	34 (6.5)	3 (0.5)	545 (100)	1742 (62)	1012 (36)	59 (2)	2813 (100)	6 (60)	4 (40)	-	10 (100)
2nd period	21 (95)	-	1 (5)	22 (100)	429 (28)	1086 (71.8)	3 (0.2)	1518 (100)	93 (3.5)	2418 (93)	93 (3.5)	2604 (100)	147 (100)	0 (0)	-	147 (100)
	p value: not apply				*p value < 0.01*				*p value < 0.01*				*p value: not apply*			

p value (Chi-square test) for proportion comparison between mild group vs (moderate + severe) group

Mild cases correspond to uncomplicated disease (severity level =1).
Moderate cases correspond to uncomplicated disease (severity level =2a).
Severe cases correspond to more complicated forms of the disease (severity level > 2b,3,4).
1st period: first half of the epidemic (Apr 2011 – 24th Feb 2012).
2nd period: second half of the epidemic (30th Mar 2012 – 31th Dec 2012).

Supplementary Table 9. Age groups of reported HFMD cases at place of admission in Hai Phong city in 2 periods of the epidemic

Age group	*1st Period of epidemic*					*2nd Period of epidemic*				
	Commune health station & At home	*District hospital*	*Hai Phong Pediatric hospital*	*Sum*	*p value*	*Commune health station & At home*	*District hospital*	*Hai Phong Pediatric hospital*	*Sum*	*p value*
under 2 years	5 (0.5)	168 (16.5)	822 (83)	995 (100)	$p = 0.261$	56 (2.7)	679 (33.3)	1305 (64)	2040 (100)	$p < 0.001$
2 years & more	5 (0.2)	377 (15.8)	2002 (84)	2384 (100)		91 (4.08)	839 (37.64)	1299 (58.28)	2229 (100)	

p value (Chi2 test)

Supplementary Table 10. Living area of reported HFMD cases at place of admission in Hai Phong city

Living area	*1st Period of epidemic*					*2nd Period of epidemic*				
	Commune health station & At home	*District hospital*	*Hai Phong Pediatric hospital*	*Sum*	*p value*	*Commune health station & At home*	*District hospital*	*Hai Phong Pediatric hospital*	*Sum*	*p value*
urban	8 (0.5)	114 (7.5)	1427 (92)	1549 (100)	$p^* = 0.261$	138 (8)	235 (13.5)	1358 (78.5)	1731 (100)	$p < 0.001$
Not urban	2 (0.1)	431 (23.5)	1397 (76.4)	1830 (100)		9 (0.35)	1283 (50.55)	1246 (49.09)	2538 (100)	

p value (Chi2 test)

*p** value (Fisher exact test)

Supplementary Table 11. Living area and severity of reported HFMD cases in Hai Phong pediatric hospital city

Living area	Hai Phong pediatric hospital									
	1st period					2nd period				
	mild	moderate	severe	*sum*	*p value*	mild	moderate	severe	*Sum*	*p value*
urban	896 (63.01)	496 (34.88)	30 (2.11)	1422 (100)	*p = 0.47*	47 (3.46)	1275 (93.89)	36 (2.65)	1358 (100)	*p = 0.02*
Not urban	846 (60.82)	526 (37.10)	29 (2.08)	1391 (100)		46 (3.69)	1143 (91.73)	57 (4.57)	1246 (100)	

Supplementary Table 12. Propagation of the HFMD epidemic among Hai Phong city districts according to median case. Rural and urban districts were differentiated (Type) and described according to major features (short + long description). Stratification (Group) was performed according to the relative order of median in the three waves (Suppl Fig 3). Date of the median case and relative order of the district was given for each waves.

Name	type	Short	long description	Group	Median W1	Wave1	Median W2	Wave2	Median W3	Wave3
Ngô Quyền	urban	HPC	Hai Phong city center	G1	24/11/2011	1	21/04/2012	11	14/10/2012	12
Hải An	urban	HPC	area encompassing airport and harbor	G1	25/11/2011	2	22/04/2012	13	14/10/2012	13
Hồng Bàng	urban	HPC	west suburb, industrial	G1	27/11/2011	4	18/04/2012	10	10/10/2012	10
Lê Chân	urban	HP	close to Hai Phong city center	G2	25/11/2011	3	16/04/2012	7	27/09/2012	5
Kiến An	urban	HP	new suburb, south	G2	29/11/2011	5	16/04/2012	8	15/09/2012	2
An Dương	Rural	W	rural, west, along tracks + big crossroad	G2	30/11/2011	6	21/04/2012	12	02/10/2012	7
Dương Kinh	urban	HP	new area close to highway	G3	01/12/2011	7	10/03/2012	1	19/09/2012	4
Đồ Sơn	urban	DS	city in the south, harbor, industry	G3	01/12/2011	8	14/04/2012	6	17/10/2012	14
Thủy Nguyên	Rural	N	rural, north	G3	03/12/2011	9	31/03/2012	3	13/10/2012	11
Kiến Thụy	Rural	E	rural, south, between Hai Phong and Đồ Sơn	G3	04/12/2011	10	23/03/2012	2	07/10/2012	8
Tiên Lãng	Rural	S	rural south	G3	07/12/2011	11	04/04/2012	4	09/10/2012	9
An Lão	Rural	W	rural, west, motorway junction	G4	09/12/2011	13	16/04/2012	9	02/10/2012	6
Vĩnh Bảo	Rural	S	rural, extreme south	G4	16/12/2011	14	05/04/2012	5	17/09/2012	3
Cát Hải	Rural	E	island	G5	07/12/2011	12	23/04/2012	14	29/08/2012	1

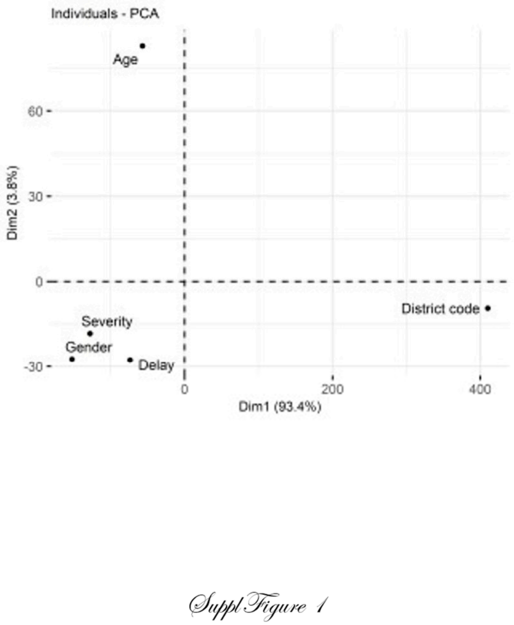

Figure S1. Spatial distribution of the Coxsackievirus-positive PCR samples during Wave 3. In-formation was confirmed by Sanger sequencing.

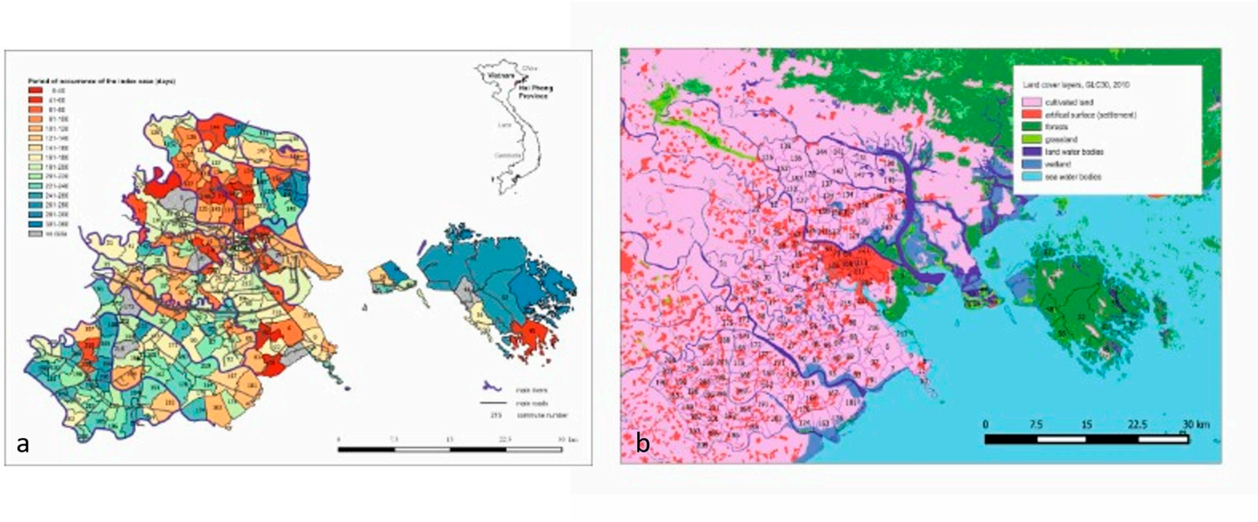

Suppl Figure 2

Figure S2. Primary component analysis. Axis 1 represented more than 95% of the variance associated with patients, based on age, gender, district related to the address, time from onset of symptoms to admission (delay), and severity.

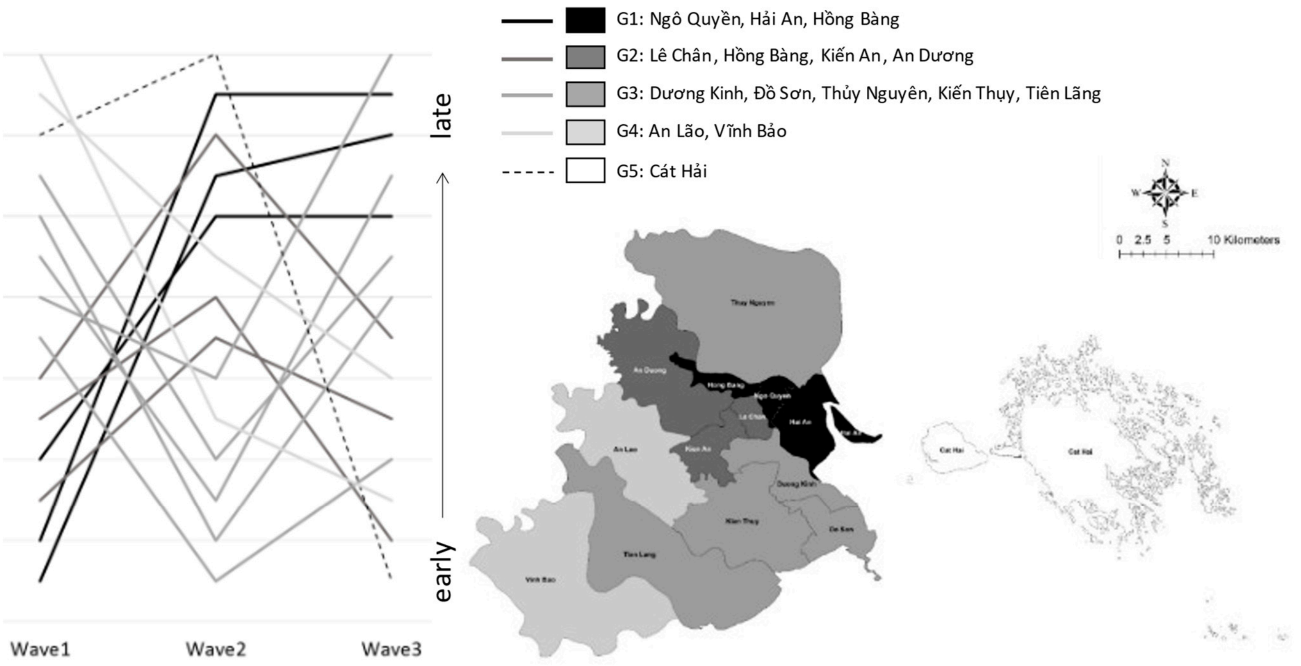

Suppl Figure 3

Figure S3. Propagation of the HFMD epidemic among Hai Phong city districts according to median case. Rural and urban districts were differentiated (Type) and described according to major features (Supplementary Table S12).

Stratification (Group) was performed according to the relative order of median in the three waves. The date of the median case and the relative order of the district were given for each wave.
